# The First, Though Belated, Account of past Dangers

**Published:** 1919-02-01

**Authors:** 


### BRITISH HOSPITALS UNDER BOMBARDMENT.

### The First, Though Belated, Account of Past Dangers.

Mercifully, deliberate specific bombing of hospitals, as experienced at Etaples and elsewhere, was not the fate of hospitals in this country during the great war. The commanders of German aircraft, whatever their intentions in this respect might have been, were never allowed by our system of defences to do more than drop their missiles haphazard, doubtless in the hope that as much general damage to property and injury to persons as possible would result. British hospitals with their helpless inmates ran terrible risks, over and over again, but came through many bombardments with but few scars and without a single instance, according to present information, of serious personal injury to the inmates.

The first recorded injury to a hospital took place on the night of June 4, 1915, as a result of a Zeppelin raid over the estuary of the Thames, when an incendiary bomb fell upon Gravesend Hospital. The present secretary, Mr. C. E. Chapman, was a spectator of the raid, in which both high explosives (used, it is believed, for the first time in an attack upon England) and incendiary bombs, which lighted up the town, were used; he hastened to the hospital and found that Block "B," which is used as night nurses' quarters, had been set on fire by an incendiary bomb, but the staff had promptly taken action with the fire appliances, and when the fire brigade arrived it was found that the fire was well in hand. There were no casualties, and altogether in Gravesend, in what was a very severe raid, there were only four people injured. A neighbouring V.A.D. hospital was damaged the same night by a high-explosive bomb bursting just outside.

There was an important Zeppelin raid over London about eleven o'clock on the night of September 8, 1915, when no fewer than five hospitals suffered, namely, St. Bartholomew's, the National Hospital for the Paralysed and Epileptic, the Italian Hospital, the London Homœopathic Hospital, and the Alexandra Hospital for Children. The first-named two institutions bore the brunt of the affray, and the last four were all affected by one bomb which fell in Queen Square. One of the bombs fell in Bartholomew Close, about twenty yards from the Little Britain side of St. Bartholomew's, and its effects can still be seen on the stonework of the hospital's northern entrance. Some 1,500 windows were broken, and, in the Nurses' Home, many windows with their frames were blown out or wrecked. Several narrow escapes of nurses from injury by falling ceilings and pieces of bomb were recorded, but not a single person connected with the hospital was killed or injured.

The Queen Square bomb of the same night dealt great havoc in the neighbourhood. The bomb fell and exploded on a lawn about 60 feet from the south-west corner of the National Hospital, where damage to the building, costing £200 to put right, resulted, as well as lesser damage to the other hospitals mentioned above. The crowds next day were very large, and a collecting-box placed outside one of the hospitals was constantly filled and emptied and brought nearly £100 to the funds. There is little doubt that had the Censor been more lenient a sympathetic public would have even more handsomely subscribed to all the hospitals concerned. But the greatest subject for congratulation was that nobody was hurt, except one patient who was the proud recipient of a minute cut on her hand caused by falling glass.

Two years later, on September 4, 1917, Charing Cross Hospital narrowly escaped destruction when a bomb from an aeroplane fell in the middle of Agar Street, almost opposite the main entrance. Practically all windows in the front of the building were broken, and in many cases were blown right in, including the sashes. The front wall of the hospital, which is covered by stucco, was badly damaged and pitted with holes, the area railings were all broken away, and the system of fire-alarm and call-bells was put out of order. Some of the débris from the front was thrown right over the building and fell into the courtyard and out-patient department at the back. In spite of all this, no one received any serious injury, although the wards were fully occupied.

The last-named hospital as well as many others received minor injuries from falling shrapnel and shell-cases on the nights of "air-raid week"—that preceding October 1, 1917, on October 31, 1917, February 27, 1918, and other occasions. On one of these nights a defective bomb fell close to the Hospital for Sick Children, Great Ormond Street, but did not explode.

There was a Zeppelin raid on October 1917, when all the glass in a North-country Poor-Law infirmary was broken, and early in December 1917 a Gotha dropped two bombs in the grounds of a general military hospital at Camberwell.

In the raid of March 1918 hospitals escaped, but two nursing homes at Maida Vale were badly injured, and there were several casualties (none very serious, we believe) amongst the inmates.

It is only within the last few weeks that precise details of air-raids have been permitted to be disclosed, so it is quite possible that the foregoing list is by no means complete. It is a matter for great thankfulness that our hospitals and their inmates have been protected against "the terror that flieth by night," and have won through, in their mission of mercy, the most terrifying years of their history.

